# Mechanistic validation of the 2016 American Society of Echocardiography/European Association of Cardiovascular Imaging Guidelines for the assessment of diastolic dysfunction in heart failure with reduced ejection fraction

**DOI:** 10.1186/s12947-020-00224-z

**Published:** 2020-10-16

**Authors:** Ythan H. Goldberg, David Megyessi, Mischa Flam, Daniel M. Spevack, Martin G. Sundqvist, Martin Ugander

**Affiliations:** 1 Department of Medicine, Albert Einstein College of Medicine-Montefiore Medical Center, Bronx, NY USA; 2 Department of Clinical Physiology, Karolinska University Hospital, and Karolinska Institutet, Stockholm, Sweden; 3 Westchester Medical Center, Valhalla, NY USA; 4 Department of Cardiology, Södersjukhuset, and Karolinska Institutet, Stockholm, Sweden; 5 Kolling Institute, Royal North Shore Hospital, and Charles Perkins Centre, Faculty of Medicine and Health, University of Sydney, Sydney, Australia

**Keywords:** Transthoracic echocardiography, Diastolic function, Pulsed-wave Doppler, Hemodynamics

## Abstract

**Background:**

The American Society for Echocardiography/European Association of Cardiovascular Imaging (ASE/EACVI) 2016 guidelines for assessment of diastolic dysfunction (DD) are based primarily on the effects of diastolic dysfunction on left ventricular filling hemodynamics. However, these measures do not provide quantifiable mechanistic information about diastolic function. The Parameterized Diastolic Filling (PDF) formalism is a validated theoretical framework that describes DD in terms of the physical properties of left ventricular filling.

**Aims:**

We hypothesized that PDF analysis can provide mechanistic insight into the mechanical properties governing higher grade DD.

**Methods:**

Patients referred for echocardiography showing reduced left ventricular ejection fraction (< 45%) were prospectively classified into DD grade according to 2016 ASE/EACVI guidelines. Serial E-waves acquired during free breathing using pulsed wave Doppler of transmitral blood flow were analyzed using the PDF formalism.

**Results:**

Higher DD grade (grade 2 or 3, *n* = 20 vs grade 1, *n* = 30) was associated with increased chamber stiffness (261 ± 71 vs 169 ± 61 g/s^2^, *p* < 0.001), increased filling energy (2.0 ± 0.9 vs 1.0 ± 0.5 mJ, *p* < 0.001) and greater peak forces resisting filling (median [interquartile range], 18 [15–24] vs 11 [8–14] mN, *p* < 0.001). DD grade was unrelated to chamber viscoelasticity (21 ± 4 vs 20 ± 6 g/s, *p* = 0.32). Stiffness was inversely correlated with ejection fraction (*r* = − 0.39, *p* = 0.005).

**Conclusions:**

Higher grade DD was associated with changes in the mechanical properties that determine the physics of poorer left ventricular filling. These findings provide mechanistic insight into, and independent validation of the appropriateness of the 2016 guidelines for assessment of DD.

## Introduction

The prevalence of HF in adults is 1–2% in developed countries, and approximately 10% among people above 70 years of age [1]. As the population of the elderly is expected to increase, HF is expected to become more prevalent in the future, placing a greater financial burden on the health care system. HF can occur in the setting of either reduced ejection fraction (HFrEF) or preserved ejection fraction (HFpEF) [1], with an approximately similar incidence and mortality risk [2]. The most common cause of diastolic dysfunction (DD) is systolic dysfunction [3]. Importantly, patients with combined systolic and DD have much poorer prognosis [4]. Hence, assessment of both systolic and diastolic function is of outmost importance.

Diastolic function assessed by Doppler echocardiography can be categorized by severity, which is related to prognosis [5–7]. In 2016 the American Society of Echocardiography and the European Association of Cardiovascular Imaging jointly updated their guidelines on the echocardiographic evaluation of left ventricular (LV) diastolic function in an effort to increase the clinical utility of diagnosing and grading severity of (DD) [8, 9]. In order to assess severity of DD, the guideline based parameters measure the effect of loading conditions of the LV (ratio of early to late mitral inflow velocities [E/A], mitral inflow velocity deceleration time, mitral annular early diastolic tissue velocity [e’], or E/e’) or sequelae of elevated left atrial (LA) pressures (LA volume index, right ventricular systolic pressure) [10]. Reduction in preload and afterload are the focus of current treatments for DD, and can be assessed by these conventional measurements, but there is no proven therapy that addresses the underlying mechanisms of DD. A challenge in developing such treatments is the lack of a practical method to measure the physical myocardial properties of diastolic function. Diastolic function itself has multiple facets, including LV relaxation, stiffness, and restoring and resisting forces, to name a few. Describing these intrinsic LV myocardial properties is not possible with conventional echocardiographic measurements [11–13] .

The parameterized diastolic filling (PDF) formalism is a method for characterizing LV diastolic function within the theoretical framework of a damped harmonic oscillator. In this model, the velocity of mitral inflow over time (the pulsed wave Doppler E-wave) is described using the same mathematics used to describe the recoil of a spring after compression. This method has been validated in prior studies in healthy subjects as well as in those with hypertension, heart failure and diabetes [14–18]. By fitting the E-wave curve to the second-order equation of a damped harmonic oscillator, the constants of stiffness (k), viscoelasticity (c), and displacement (x_0_) can be determined [19].

The aim of the study was to apply the PDF formalism to study LV diastolic function from a mechanistic standpoint in a real-world sample of patients with HFrEF and determine whether patients with different DD grades display different mechanistic properties of diastolic function.

## Methods

### Subject selection

**Fig. 1:**
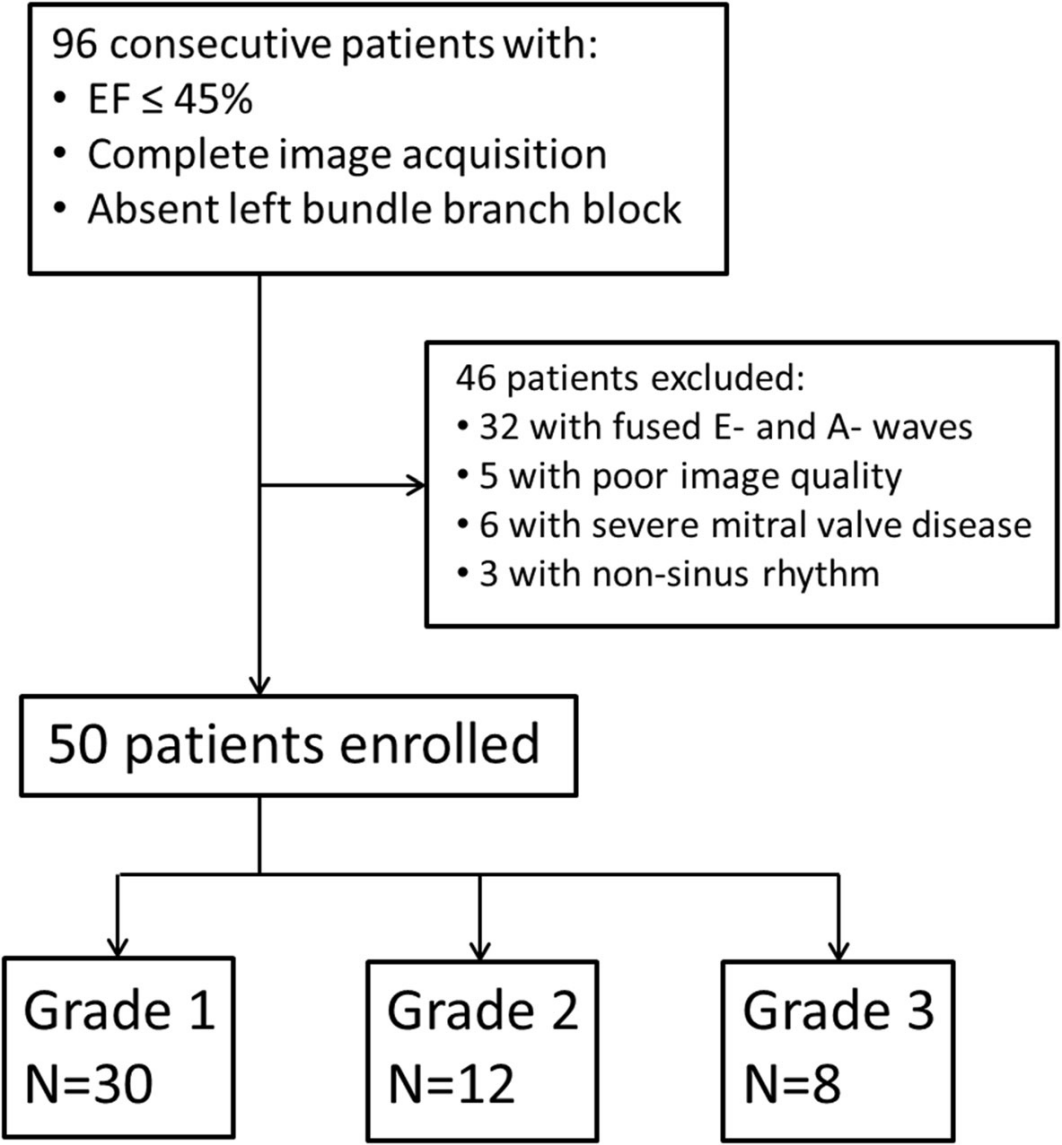
A schematic flowchart illustrating the patient selection process

After obtaining local institutional review board approval, clinical transthoracic echocardiograms performed by three experienced technicians at the two primary teaching hospitals of Montefiore Medical Center between October 2014 and January 2015 were prospectively recruited. The study’s original goal was to provide mechanistic validation of the 2009 ASE/EACVI recommendations for the evaluation of left ventricular diastolic function by echocardiography [8]. However, after completion of data collection the 2016 ASE/EACVI guidelines were published, and data analysis was performed according to the updated definition and grading criteria for diastolic dysfunction [9]. Studies were included for analysis if the EF was less than or equal to 45% in a native heart without being on vasopressor, inotropic or mechanical support, and in the presence of sinus rhythm without left bundle branch block. The 45% EF cutoff was chosen because standard of care medical therapy for systolic heart failure is routinely administered at 45% and below. Studies were excluded if there was severe mitral valvular disease, mitral calcification restricting mitral inflow, or fusion of E- and A-waves. A flow chart describing patient selection is presented in Fig. 1.

### Echocardiographic data acquisition

Examinations were carried out with subjects in the standard left lateral position. From the apical four-chamber view, pulsed-wave Doppler acquisitions of transmitral blood flow velocities with approximately 20 consecutive E-waves were recorded during free breathing. All images were acquired with a clinical echocardiography system (Philips iE33, Philips Medical Systems, Andover, MA, USA) with an S5–1 probe, 2.5 MHz. Wall filter was set at 125 Hz. To visualize E-waves in their entirety, baseline and velocity were optimized, and horizontal sweep was set to 100 mm/s. Transmitral flow parameters used for conventional grading of DD, such as E, A, lateral E/E’, LA volume and E-wave DT, as well as heart rate and EF, were measured by experienced cardiologists board-certified in echocardiography. Doppler examinations were saved in the digital imaging and communications in medicine (DICOM) format.

### Analysis of E-waves using the PDF formalism

**Fig. 2:**
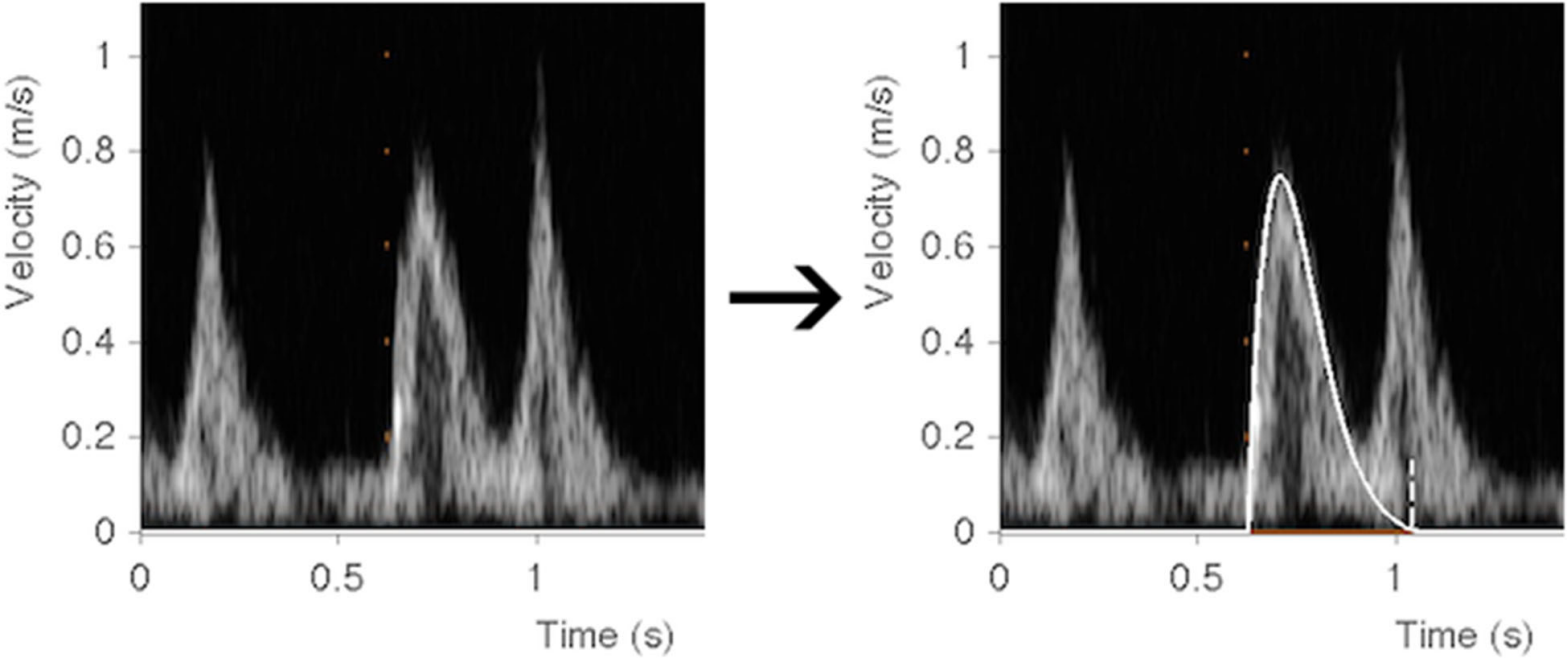
The figure shows an E-wave from pulsed-wave Doppler measurement of the flow across the mitral valve. The solid white line (right panel) shows the curve fit of the PDF method whereby the mathematical formula describing a damped harmonic oscillator is fit to the contour of the E-wave, thus generated the PDF measures. Analysis was performed using the freely available software www.echoewaves.org

Images were analyzed using freely available and validated software for PDF analysis (Echo E-waves, echoewaves.org) [20]. The program’s feature for edge detection estimated the curve describing the velocity envelope of each E-wave, and manual adjustments were made to optimize fit with the Doppler signal contour (see Fig. 2). Because this program version could only perform E-wave analysis on DICOM files, mitral inflow Doppler envelopes either at the beginning or end of the acquisition image frame could not be used. As a result, out of the twenty cardiac cycles 10–15 were used for analysis. Values were generated for the following PDF parameters: viscoelasticity (c), stiffness (k), load (x_0_), kinematic filling efficiency index (KFEI), damping index (β), peak resistive force of filling (kx_0_), peak driving force of filling (cE_peak_), filling energy (1/2kx^2^), the load-independent index of diastolic filling (M), the peak driving force at zero peak resistive force, (intercept B), and the estimated time constant of isovolumetric pressure decay (tau). These measures have been described and validated previously. These measurements, which are summarized in Table 1, were compared across DD grades as described in the 2016 ASE/EACVI guidelines for evaluation of diastolic dysfunction. Ten patients were randomized to intra- and inter-observer variability evaluation, respectively.

**Table 1:** Explanation of PDF paramaters and indices

PDF Parameter or Index	Physiologic Analog	SI Units	Expressed as	Description
**x**_**0**_	Effective volumetric load	cm	cm	Initial displacement, effectively equivalent to the E-wave VTI
**c**	Viscoelastic loss index	N·s·m^−1^	g/s	Friction-like force which opposes the ventricle returning to its resting state after systole
**k**	Chamber stiffness	N·m^−1^	g/s^2^	Analogous to the spring stiffness constant, and correlated with invasive LV dP/dV
**kx**_**0**_	Maximum driving force	N	dynes or mN	The initial peak driving force of diastole. Proportional to the peak atrioventricular pressure gradient
**1/2kx**_**o**_^**2**^	Potential energy	J	ergs or mJ	Stored potential elastic energy to generate rapid recoil during early filling
**cE**_**peak**_	Peak resistive force	N	mN	The initial peak resistive force of diastole. Resistive (viscoelastic) force at peak flow
**M**	Load independent index of diastolic filling	N/N	unitless	Unitless ratio of maximum driving force to peak resistive force (kx_0_/cE_peak_)
**c**^**2**^**-4k**	Damping index β		g^2^/s^2^	Relative contribution between damping (c) and recoil (k). Negative values reflect underdamped filling, positive values reflect overdamped filling
**KFEI**	Kinematic filling efficiency index	cm/cm	unitless	Ratio of the VTI of the acquired E-wave contour fit via PDF to the VTI of the PDF model-predicted ideal E-wave contour with no resistance to filling (c = 0)
**Slope intercept B**	Maximum driving force for cE_peak_ = 0	N	mN	y-intercept of the equation: kx_o_ = M·cE_peak_ + B; peak driving force in the setting of no resistance, related to LVEDP

*VTI* velocity time integral, *LVEDP* left ventricular end-diastolic pressure

### Statistical analysis

Unless otherwise stated, statistical analysis was performed using the software SPSS (Version 24, IBM, Armonk, New York, USA). The Kolmogorov-Smirnov test was used to test for normality of distribution for continuous variables. Normally distributed variables were compared between groups of diastolic dysfunction grade with the two-tailed Student’s t-test and reports as mean ± standard deviation. For non-normally distributed data, groups were compared using the independent samples Mann-Whitney U-test expressed as median [interquartile range]. Categorical data was presented as number and percentage. A *p* value < 0.05 was taken as the limit for statistical significance. Pearson’s correlation coefficient was calculated for linear regression analysis. For inter- and intraobserver reliability analysis, respectively, intra-class correlations were estimated using Stata 15 (StataCorp, College Station, TX) based on absolute agreement for single measurements using two-way random and mixed effects models.

## Results

Applying the 2016 ASE/EACVI guidelines to the echocardiographic evaluation of diastolic function [9], patients were classified as DD grade 1 (*n* = 30) or grade 2–3 (*n* = 20) (age 64 ± 14 years, 62% male, heart rate 73 ± 14 beats/min, no differences between groups). Patient demographic and conventional echocardiographic data are described in Table 2. Left ventricular ejection fraction (LVEF) was lower, and left atrial volume index (LAVI) and left ventricular mass index (LVMI) were higher in the high DD grade group. While E was also higher in the high DD grade group, A and lateral e’ did not differ between groups.

**Table 2:** Patient characteristics

	Diastolic dysfunction grade 1	Diastolic dysfunction grade 2 or 3	***p***-value
	(***n*** = 30)	(***n*** = 20)	
**Age, years**	62 ± 15	68 ± 14	0.15
**Females, n (%)**	11 (37)	8 (40)	0.81
**Hypertension, n (%)**	19 (63)	19 (95)	0.01
**Diabetes Mellitus, n (%)**	12 (40)	14 (70)	0.04
**Ischemic Cardiomyopathy, n (%)**	20 (67)	11 (55)	0.41
**Chronic Kidney Disease**^a^**, n (%)**	5 (17)	5 (25)	0.47
**Heart rate, beats/min**	73 [63, 75]	74 [62, 85]	0.33
**LVEF, %**	40 [35, 45]	35 [29, 41]	**0.04**
**LAVI, ml/m2**	25.2 ± 5.9	39.3 ± 5.9	**< 0.001**
**LVMI, g/m2**	114 ± 30	137 ± 40	**0.03**
**LVEDD, mm**	53 [46, 58]	57 [52, 60]	0.06
**E, cm/s**	65 [54, 74]	106 [95, 128]	**< 0.001**
**A, cm/s**	77 [63, 89]	63 [43, 3]	0.07
**E/A ratio**	0.88 [0.66, 1.05]	2.00 [1.50, 2.80]	**< 0.001**
**DT, ms**	206 ± 49	161 ± 50	**0.003**
**e’ lateral, cm/s**	7.3 ± 2.5	6.4 ± 2.0	0.16
**E/e’ lateral ratio**	9.8 [7.0, 11.8]	18.1 [14.0, 19.5]	**< 0.001**

Data are presented as mean ± SD or median [interquartile range]. *LVEF* left ventricular ejection fraction, *LAVI* left atrial volume index, *LVMI* left ventricular mass index, *LVEDD* left ventricular end-diastolic diameter, *DT* deceleration time. ^a^denotes at least moderate chronic kidney disease (CKD) stage (3A or higher) as defined by the National Kidney Foundation

**Fig. 3:**
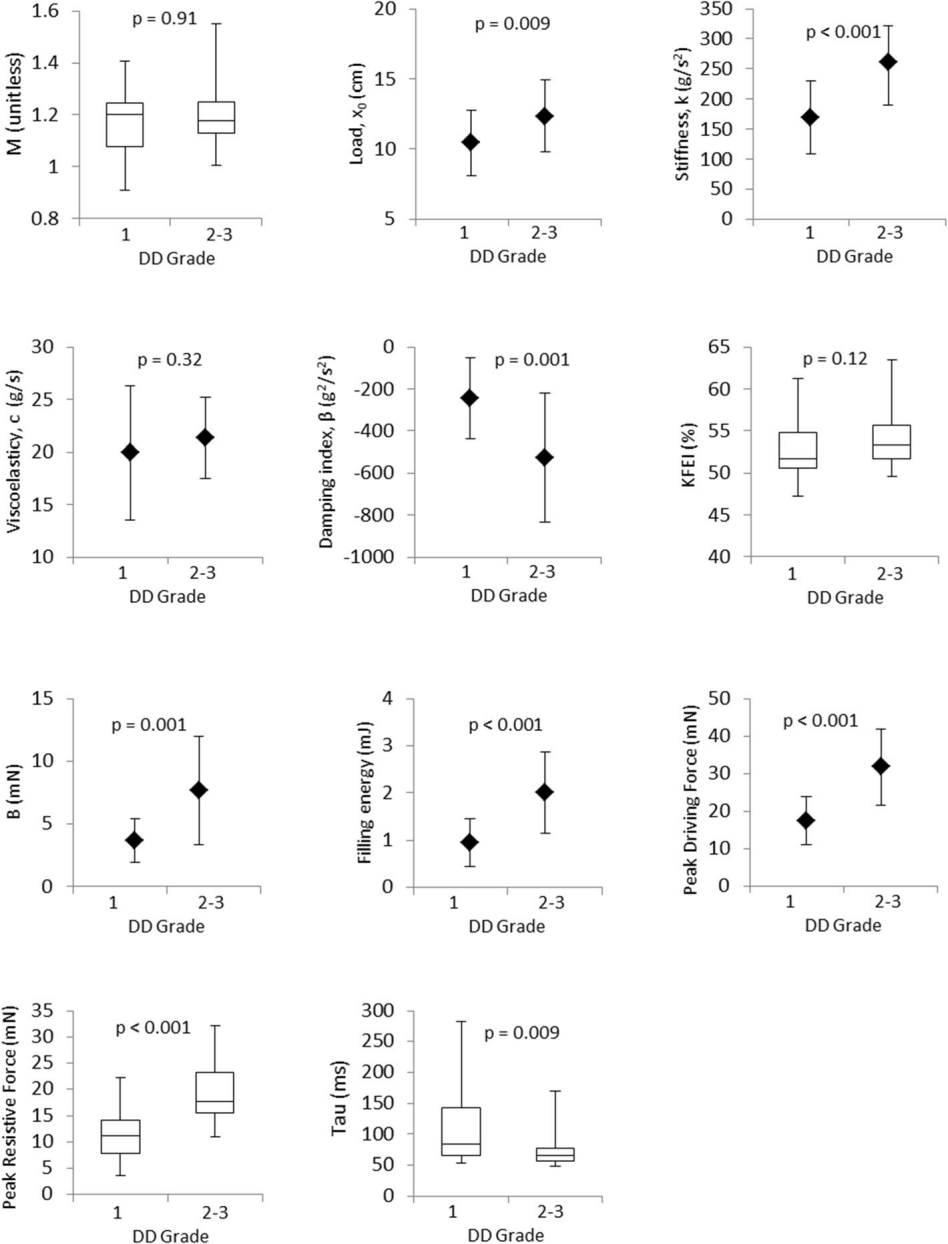
Results of the PDF analysis according to grade of diastolic dysfunction (DD)

Comparison of the PDF parameters between groups is displayed in Fig. 3. Higher DD grade was associated with increased initial load, x_0_ (12.3 ± 2.6 vs. 10.4 ± 2.3 cm, *p* = 0.009), and chamber stiffness, k (261 ± 71 vs. 169 ± 61 g/s^2^, *p* < 0.001), while viscoelasticity, c, did not differ between the groups (21 ± 4 vs. 20 ± 6 g/s, *p* = 0.32). As a consequence, the high DD group had a higher filling energy (2.0 ± 0.9 vs. 1.0 ± 0.5 mJ, *p* < 0.001) and peak driving force of filling (31.7 ± 10.0 vs. 17.5 ± 6.4 mN, *p* < 0.001). Similarly, the damping index β was more negative in the high DD group, indicating greater damping relative to recoil (− 245 ± 195 vs. -526 ± 308 g^2^/s^2^, *p* = 0.001). The high DD group had a greater peak resistive force of filling (19.8 [15.6, 23.2] vs. 11.7 [7.8, 14.0] mN, *p* < 0.001), and this can be attributed to a greater atrioventricular pressure gradient as denoted by higher E-wave maximum velocity (106.2 [95.2, 127.8] vs. 65.1 [54.1, 74.2] cm/s, *p* < 0.001). By comparison, the higher peak resistive force was not due to greater intrinsic damping properties within the ventricle as denoted by the lack of difference in viscoelasticity. The predicted peak driving force at zero resistive force (B intercept) was also increased in the group with high DD grade (7.65 ± 4.31 vs. 3.71 ± 1.76 mN, *p* = 0.001). There were no differences between the two groups with regards to the load-independent index of filling, M (1.20 [1.13, 1.25], vs. 1.17 [1.08, 1.25], *p* = 0.91) or the kinematic filling efficiency index (54.0 [51.6, 55.6] vs. 52.7 [50.5, 54.8] %, *p* = 0.12). Curiously, tau was lower in the high DD group (65 [57, 77] vs. 83 [66, 143], *p* = 0.009). The stiffness constant k was inversely correlated with ejection fraction (*r* = − 0.39, *p* = 0.005).

All parameters had good to excellent intra-observer variability (intraclass correlation coefficient 0.69 to 0.95). Inter-observer variability was good to excellent for all parameters except the derived parameters KFEI, M, and β.

## Discussion

The unique finding of this study is that higher grade DD in the setting of reduced LVEF was associated with non-invasively quantifiable changes in the mechanical properties that determine the physics of poorer left ventricular filling. Specifically, more severe DD was associated with a higher load, greater ventricular stiffness, higher energy of filling, and greater peak driving and peak resistive forces. These findings provide mechanistic insight into, and independent validation of the appropriateness of the 2016 ASE/EACVI guidelines for assessment of DD.

Mechanistic validation of these guidelines in the HFrEF population to date is limited. DD grade has been shown to not be associated with prolonged Tau (> 48 ms) in patients with systolic LV dysfunction, though there was an association between DD grade and left ventricular end diastolic pressure (LVEDP) [21]. The E/A ratio was shown to be the only independent parameter correlated with LVEDP in 39 subjects with EF < 50% [22]. In that study, overall accuracy of the guideline-derived algorithm to identify LVEDP > 15 mmHg was shown to be modest, with a sensitivity of 61% and specificity of 69%. In contrast, the algorithm accurately diagnosed elevated LVEDP in 91% of subjects with EF ≤35% [23]. There are several possible explanations for the discrepancy between these two prior studies. Compared to the latter study, the former excluded individuals with acute coronary syndrome, decompensated heart failure, and more than mild valvular disease. Mean LVEDP was 16 mmHg in the low EF subgroup of the former study, versus 21 mmHg in the latter study (LVEDP not specified for the low EF subgroup). Further insights may be obtained from future studies comparing PDF measurements with invasive hemodynamic data.

The relationship between E-wave velocity and diastolic function is represented by a J-shaped curve: tall E-waves in the setting of a high atrioventricular pressure gradient due to strong ventricular suction in normal diastolic function, short E-waves when relaxation is impaired but atrial pressure is normal, and tall E-waves again with impaired relaxation and elevated atrial pressure. The E-wave itself is a sum of several processes, namely, the load placed on the elastic elements during contraction as well as their stiffness, and early relaxation due to actin-myosin cross-bridge uncoupling. Measuring peak E-wave velocity alone therefore does not allow for the evaluation of the components of diastolic function. Other measurements such as E-wave deceleration time, early diastolic mitral annular velocity, and transmitral flow propagation velocity provide limited mechanistic insight, but the PDF formalism allows for noninvasive assessment of ventricular relaxation with considerably greater detail and with the added benefit of providing mechanistic understanding with regards to the physical properties of diastolic filling.

The current study found no difference in the load-independent index of diastolic filling (M) between our low and high DD groups. This index has been previously shown to be higher in a cohort with LVEF > 60% and LVEDP > 19 mmHg compared to normal controls [14]. By comparison, the mean value of M in the current study was similar to those in the previously published cohort with elevated LVEDP, reinforcing the concept that individuals with reduced LV systolic function have impaired mechanics of diastolic filling regardless of LVEDP [14]. The current study showed no difference in viscoelasticity between the two DD groups. The viscoelasticity constant c correlates with forces that counter elastic relaxation of the left ventricle, and has been shown to be increased in the setting of diabetes, hypertension and increasing age [24–26]. The current study had a relatively high overall incidence of diabetes (52%), and the mean value of c in the current study was between the values previously reported for patients with and without diabetes [19]. In light of those findings, this lack of a difference in viscoelasticity between the low and high DD groups is expected.

Compared to the low DD grade cohort, the high DD group had higher stiffness (k), lower time-constant of isovolumic relaxation (tau), and shorter deceleration time. Prior work has shown patients with normal LVEF can have increased invasively determined end-diastatic chamber dP/dV stiffness [15], and it is known that stiffness by the PDF method correlates strongly with the average **Δ**P/**Δ**V measure of stiffness as determined by invasive measurements [27]. Given that viscoelasticity was similarly impaired in both groups in the current study, it stands to reason that chamber stiffness and not viscoelasticity is the main determinant of increased left atrial pressure in the setting of reduced LVEF. Indeed, we found a negative correlation between k and LVEF. This is notable because neither diabetes, hypertension, nor age have been associated with increased stiffness as measured by PDF parameters to date [24, 25, 28]. Invasively determined LV myocardial diastolic stiffness has been shown to be increased in the setting of both HFpEF and HFrEF, but only those with reduced LVEF had significantly higher myocardial collagen content and increased cardiomyocyte resting tension compared to patients without diabetes [29]. In addition to the extracellular matrix, intracellular changes in titin inherent to the pathophysiology of reduced systolic function may also affect stiffness [30]. Contractility itself, however, has not been shown to affect left ventricular stiffness [31]. Stiffness has been shown to be increased in coronary artery disease, and even more so in the setting of acute myocardial infarction [32]. The majority of patients in this study had ischemic cardiomyopathy. Individuals with HFrEF exhibit numerous changes within the cardiovascular system, namely systemic and cardiac inflammation, endothelial dysfunction, eccentric cardiac hypertrophy, cardiomyocyte apoptosis, and extracellular cardiac fibrosis [33]. All these processes speak to the complexity and multifactorial aspects that determine LV diastolic function.

### Limitations

The current study has several limitations that deserve to be acknowledged. Though our measurements were not directly compared to invasively derived data, these PDF methods have been validated in prior studies [19, 26, 27]. The sample size in the current study is relatively small, and could be underpowered to demonstrate differences for some parameters. Likewise, there were not enough patients with DD grade 3 to determine whether this subgroup could be further differentiated from lower DD grades by PDF measurements. Our results demonstrate how relaxation is impaired in left ventricles with systolic dysfunction but cannot be generalized to those with normal systolic function, and such studies would be of value to undertake. Likewise, we did not explore differences in diastolic dysfunction between ischemic and non-ischemic cardiomyopathy. PDF analysis is limited to the phase of early diastolic filling and does not account for the period of active atrial contraction. The current ASE/EACVI guidelines for assessing diastolic function are clinically useful for estimation of left atrial pressure. The PDF formulism is meant to perform in a complimentary role to traditional diastology.

### Conclusions

In conclusion, higher grade DD was associated with a deterioration of the mechanical properties that determine the physics of left ventricular filling. These findings provide mechanistic insight into, and independent validation of the appropriateness of the 2016 guidelines for assessment of DD.

## Data Availability

The data that support the findings of this study are available from the corresponding author upon reasonable request.
